# Association Between Inter-Limb Asymmetry and Determinants of Middle- and Long-distance Running Performance in Healthy Populations: A Systematic Review

**DOI:** 10.1186/s40798-024-00790-w

**Published:** 2024-11-26

**Authors:** Joachim D’Hondt, Laurent Chapelle, Chris Bishop, Dirk Aerenhouts, Kevin De Pauw, Peter Clarys, Eva D’Hondt

**Affiliations:** 1 Movement and Nutrition for Health and Performance (MOVE) Research Group, Department of Movement and Sport Sciences, Faculty of Physical Education and Physiotherapy, Vrije Universiteit Brussel, Pleinlaan 2, 1050 Brussels, Belgium; 2 London Sport Institute, Middlesex University, London, UK; 3 Human Physiology and Sports Physiotherapy (MFYS) Research Group, Department of Physiotherapy, Human Physiology and Anatomy, Faculty of Physical Education and Physiotherapy, Vrije Universiteit Brussel, Brussels, Belgium; 4 Brussels Human Robotics Research Center (BruBotics), Vrije Universiteit Brussel, Brussels, Belgium

**Keywords:** Side-to-side differences, Bilateral difference, Between-limb, Functional asymmetry, Morphological asymmetry, Kinematic asymmetry, Kinetic asymmetry, Biomechanics, Running economy, Distance running

## Abstract

**Background:**

The presence of inter-limb asymmetry in the human body has traditionally been perceived to be detrimental for athletic performance. However, a systematic review addressing and comprehensively assessing the association of asymmetry between the lower limbs and middle- and long-distance running performance-related metrics is currently lacking.

**Objective:**

The main purpose of this systematic review was to examine the relationship between lower inter-limb asymmetry and determinants of running performance in healthy middle- and long-distance runners. The secondary objective was to identify possible avenues for further research in this area.

**Methods:**

PubMed, Web of Science and SPORTDiscus were systematically searched for studies investigating the relationship between lower inter-limb asymmetry and (determinants of) running performance in healthy and injury-free middle- and long-distance runners. The quality of studies eligible for inclusion was assessed using the Downs and Black Quality Index Tool.

**Results:**

Out of 4817 articles screened, 8 studies were included in this review which assessed the association between functional, morphological, kinematic and kinetic asymmetry and running performance-related metrics. The quality score of the included research varied between 5/10 and 9/10. Our results revealed mixed findings, showing both significant negative (n = 16) and positive (n = 1) associations as well as no significant associations (n = 30) between inter-limb asymmetry and running performance-related metrics.

**Conclusions:**

A high heterogeneity across study methods and outcomes was apparent, making it difficult to draw a straightforward conclusion. Our results indicate that the majority of metrics of functional, morphological, kinematic and kinetic inter-limb asymmetry are negatively or not associated with running performance (and/or its determinants). Thus, a more extensive high-quality body of research using standardised asymmetry magnitude metrics is essential to determine whether, and to what extent asymmetry between the lower limbs could affect middle- and long-distance running performance. Future studies should establish potential trade-off values to help practitioners develop evidence-based training programs.

**Key Points:**

- In the majority of the metrics, the magnitude of lower inter-limb asymmetry was negatively or not associated with middle- and long-distance running performance.
- Coaches, athletes and researchers should be attentive of the task, time- and metric-specificity as well as the inter- and intra- individual variability of magnitude outcomes, when assessing inter-limb asymmetries.

## Background

The concept of lateralization is a fundamental aspect of human neurodevelopment that involves the preferential use of one side of the body over the contralateral side during voluntary movement [1]. This phenomenon, that initiates before birth and expands during early infancy, occurs in almost every individual resulting in imbalances between body sides [1]. Such inter-limb asymmetry can manifest itself in multiple dimensions, encompassing functional (e.g., strength, power, speed, range of motion and agility), morphological (e.g., muscle mass, bone mineral content and fat mass), kinematic (e.g., body centre of mass displacements, joint angles or spatiotemporal measures, such as contact time, flight time, step length and step frequency), and kinetic (e.g., peak vertical ground reaction force) measures [2–5]. Interestingly, previous studies have shown that these different types of asymmetry are not necessarily related to each other [2, 6]. Furthermore, the magnitude of asymmetry has also been reported to be specific according to the task, metric, test occasion, and individual [7–13]. Given that the presence of inter-limb asymmetry has intuitively been considered to increase injury risk and to compromise athletic performance among sport practitioners, an abundance of research has been conducted on this topic over the last decade [4, 14–16].

From a sports performance perspective, recent research has shown that a larger magnitude of functional asymmetry can be associated with impaired sport performance [17–21]. For instance, larger inter-limb differences resulting from the unilateral countermovement (CMJ) and drop jump (DJ) tests have been shown to be positively correlated (i.e., indicating poorer performance) with sprint time (CMJ: *r* = 0.43 to 0.71, DJ: *r* = 0.52 to 0.58) and change of direction time (CMJ: *r* = 0.61 to 0.71, DJ: *r* = 0.52 to 0.66) in youth team-sport athletes [20–23]. However, there are also several studies demonstrating no meaningful relationship between functional inter-limb asymmetry and athletic performance [22, 24–26]. In terms of morphological asymmetry, the existing literature on athletes shows that a higher degree of lean mass asymmetry between the lower limbs is also related to a decreased sport performance, in tasks such as kicking (*r* = − 0.31 to 0.41) [27]. Further to this, inter-limb asymmetry in calf girths has been negatively associated with cycling performance (*r* = − 0.46) [28], whereas asymmetries in knee and ankle widths have been reported to explain 5% of the variation in track and field performances [29]. Also regarding kinematic and kinetic asymmetry, several studies demonstrated significant relationships with sport performance [30, 31]. For instance, Rannama et al. [31] documented negative associations between peak isokinetic torque asymmetry (at 180° sec^−1^) (*r* = − 0.50) as well as trunk (*r* = − 0.65) and pelvis (*r* = − 0.63) kinematic asymmetry and power output during a 5-s maximal cycle test.

Although extensive research has recently been conducted on the link between inter-limb asymmetry and sports performance, the available body of literature has focused mostly on unilateral dominant sports (i.e., sports that involve primarily one side of the body or predominantly use one limb, such as tennis, soccer or cricket) [4, 32, 33]. Examining inter-limb asymmetry in unilateral dominant sports is relevant because athletes performing unilateral dominant sports will likely exhibit larger magnitudes of inter-limb asymmetry due to the demands and nature of their specific sport [2, 3]. However, research has indicated that alternating unilateral sports (e.g., cycling and running) significant inter-limb asymmetries may also occur due to the preferential use of one side of the body during repetitive movement patterns [16]. For example, functional, kinematic and kinetic asymmetries at lower limb level, respectively ranging from 16 to 17, 3 to 54% and 3 to 54%, have been reported in adult endurance runners [9, 34, 35].

In line with research focussing on unilateral dominant sports, inter-limb asymmetry could also affect running performance-related metrics. For instance, a positive relationship (*r* = 0.85) between inter-limb functional asymmetry and running economy (i.e., a major determinant of long-distance running performance) was documented in female adolescent elite middle- and long-distance runners, indicating that inter-limb asymmetry could possibly impair running performance [36]. Also, regarding morphological asymmetry, Jamaican track athletes have been shown to perform better in 100 m sprinting when having more symmetrical knee and ankle widths [29, 37]. Whilst it is acknowledged that these are non-modifiable factors, such data could have a role to play as part of the talent identification process. In contrast, previous research has shown no significant associations between maximal sprint velocity and running velocity with spatiotemporal, kinematic and kinetic asymmetry [5, 38].

Middle- and long-distance running is a popular sport and leisure time activity on a global level. Given the potential impact of inter-limb asymmetry on middle- and long-distance running performance, a clear overview of the available literature is warranted and should help practitioners better understand the role of inter-limb asymmetry in their sport. However, a systematic review on the association between inter-limb asymmetry and middle- and long-distance running performance-related metrics is currently lacking. Therefore, the main aim of this systematic review was to comprehensively synthesize and appraise the available literature relating to inter-limb asymmetry and its association with determinants of middle- and long-distance running performance in healthy populations. Based on the resulting synopsis, some possible avenues for future research on the topic will be suggested.

## Methods

This systematic review was written in accordance with the “Preferred Reporting Items for Systematic reviews and Meta-Analyses” (PRISMA) guidelines [39], and was registered on PROSPERO on 31 October 2023 (ID: CRD42023474606).

### Eligibility Criteria

Table 1 presents an overview of the eligibility criteria that were used during the systematic search in order to guide the selection procedure. All criteria were determined a priori in accordance with the population (P), outcome (O) and study design (S) dimensions from the PICO(S) acronym [39]. The study selection, data collection process and risk of bias assessment were performed in a blinded and standardized manner by two independent researchers (J.D. and L.C.). Any divergence between both reviewers was resolved by consensus, or by discussion with a third reviewer (D.A.).

**Table 1:** Eligibility criteria

	Inclusion criteria	Exclusion criteria
Participants	Healthy injury-free male and/or female participants of any age Any type of middle- and long-distance runners (e.g., distance runners, but also triathletes or duathletes) of any level	Physical conditions that may influence running asymmetry or running performance Sprint athletes (i.e., up to distances of 400 m)
Outcome of interest	Analysis of the association between lower inter-limb asymmetry and middle- and long-distance running performance (and/or its determinants)	Analysis of the association between inter-limb asymmetry and sports performance, but not specifically related to middle- and long-distance running Only reporting the magnitude of asymmetry
Study type and design	English original peer-reviewed articles No restrictions were imposed on the type of study design (e.g., cross-sectional, longitudinal and interventional studies)	Umbrella reviews, systematic reviews or meta-analyses, books, conference abstracts

### Search Strategy

**Fig. 1:**
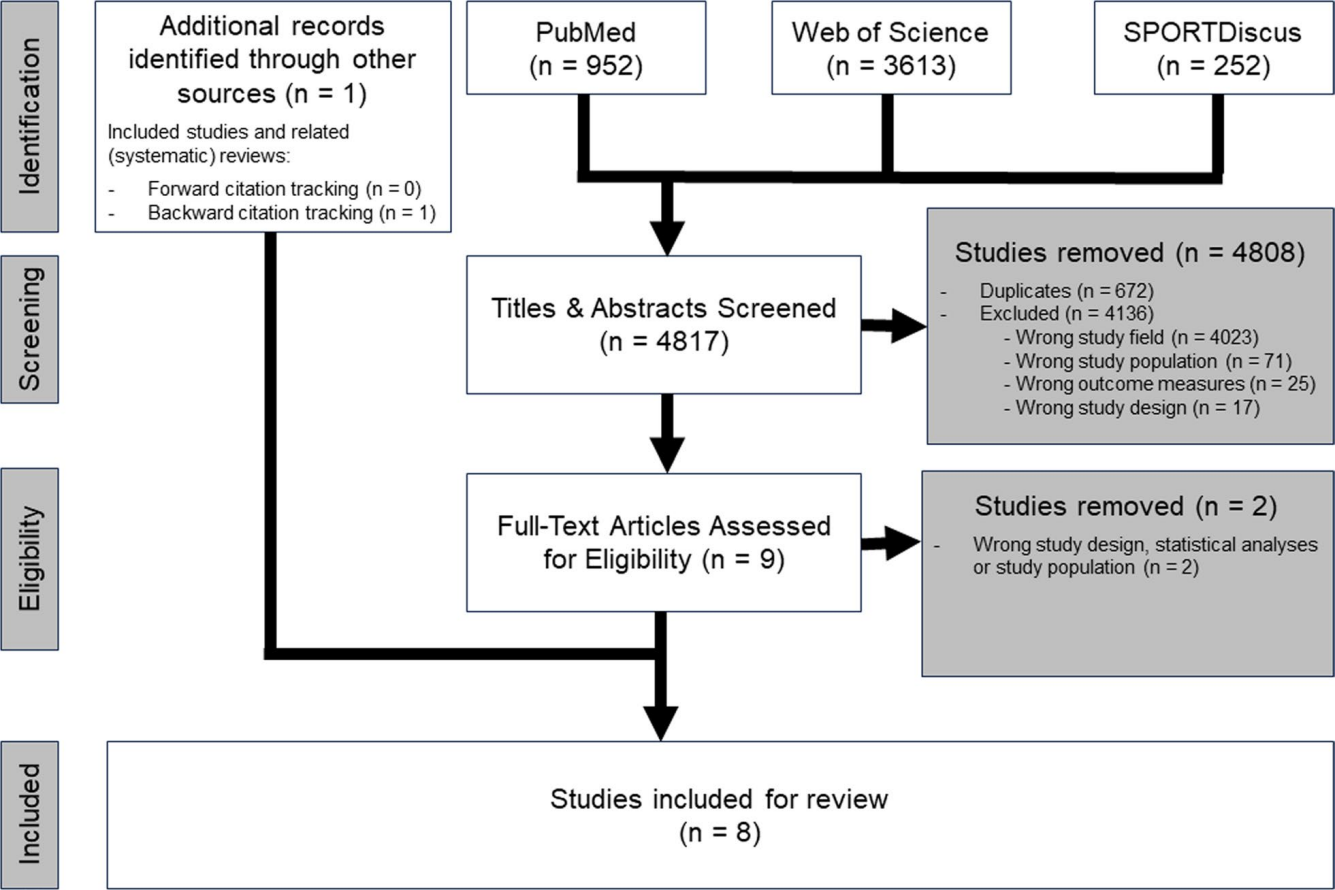
Flow diagram representing the selection and identification process of studies

The electronic databases PubMed, Web of Science, and SPORTDiscus (EBSCOhost) were systematically searched to gather relevant literature in August 2023. Searches included all papers using “Title / Abstract” for PubMed, and “Abstract” for Web of Science and EBSCOhost. In accordance with the eligibility criteria, the search was limited to English-language articles only. Additionally, “Article” and “Early access” in Web of Science, and “Academic Journals” in EBSCOhost were selected as source type, whereas no source type could be selected in PubMed. No limit was imposed on the publication date. Forward citation tracking (i.e., identifying more recent articles that cited the studies included in the present systematic review, using Web of Science) as well as backward citation tracking (i.e., screening the reference list of the studies included in the present systematic review) was performed for the eligible articles. Citations and reference lists of related (systematic) reviews identified during the search were also screened. The corresponding flow diagram is depicted in Fig. 1 and the complete search strategy with Boolean operators is detailed in Table 2.

**Table 2:** Schematic overview of the search strategy

Database	Retrievals	Complete search strategy using Boolean operators (as applied across all databases, N = 3)
PubMed Web of Science EBSCOhost	*952* *3613* *252*	(“distance runn*” OR “endurance runn*” OR “middle distance runn*” OR “jogging” OR “marathon” OR “trail runn*” OR “ultramarathon” OR “track and field” OR “5 k” OR “10 k” OR “run” OR “runn*” OR “sprint” OR “treadmill”) AND (“asymmetr*” OR “symmetr*” OR “imbalance” OR “side-to-side” OR “dissymmetr*” OR “interlimb” OR “inter-limb” OR “between-limb” OR “bilateral difference”) AND (“performance” OR “time trial” OR “trial” OR “speed” OR “velocity” OR “economy” OR “cost of running” OR “energy cost” OR “VO2max” OR “VO2peak” OR “maximal oxygen uptake” OR “oxygen consumption” OR “fatigue” OR “exhaustion” OR “lactate” OR “aerob*” OR “anaerob*” OR “stride length” OR “step length” OR “contact time*”) NOT (“injur*” OR “ACL” OR “anterior cruciate ligament” OR “syndrome” OR “traum*” OR “facture” OR “illness” OR “disease” OR “amput*” OR “stroke” OR “cerebral palsy” OR “tremor” OR “diagnosed” OR “disorder” OR “osteoarthritis” OR “geriatric” OR “return to sport” OR “rehabilitat*” OR “diagnosis” OR “pathology” OR “surgery”)

### Study Selection

All articles were retrieved from the three scientific databases consulted, and duplicates were removed using the Endnote software. Subsequently, all titles and abstracts were screened using the Rayyan software [40]. The remaining articles were evaluated based on full text and reasons for exclusion were registered.

### Data Collection Process

A self-created form was used to collect the following data per included study: year of publication, study design, sample characteristics (i.e., sample size, mean age, sex distribution, training status [e.g., athletes or non-athletes]), type of assessments as well as the metrics used to determine inter-limb asymmetry and equations applied to calculate asymmetry magnitude, association(s) between inter-limb asymmetry and any measure or metric to express participants’ running performance.

### Risk of Bias Assessment

The included studies quality was assessed using the Downs and Black Quality Index Tool [41]. In accordance with two recent systematic reviews, a modified version of this tool was used by only including the items deemed relevant for this current review [42, 43]. More specifically, the items relating to patient treatment, training interventions and group randomization processes were excluded from the assessment. Each remaining item (N = 10, see Table 3) was scored either a 1 (yes = ‘•’), a 0 (no = ‘○’) or was indicated as ‘-’ when unable to determine a score based on the information in the study reports.

**Table 3:** Questions from the modified Downs and Black [44] checklist used to evaluate methodological quality of the included studies

Item number	Question
*Reporting*	
1	Is the hypothesis/aim/objective of the study clear?
2	Are the main outcomes to be measured clearly described in the introduction or methods section? *Information outlined in introduction/methodology for both physical characteristics and running performance measure used for associative analysis pertaining to assessment(s) used, any calculations used and units of measurement
3	Are the characteristics of the subjects included in the study clearly described? *Source defined, with characteristics included
4	Are the main findings of the study clearly described?
5	Does the study provide estimates of the random variability in the data for the main outcomes? *One of the following included for both physical characteristics and running performance measures: a) mean ± *SD, b) standard error, c) confidence intervals and d) interquartile range*
6	Have actual probability values been reported (e.g. 0.035 rather than < 0.05) for the main outcomes except where the probability value is less than 0.001? *Exact correlation (r) and significance (p) values provided, specific to the associative analysis
*External validity*	
7	Were the subjects to participate in the study representative of the entire population from which they were recruited? * Proportion of subjects asked to participate, relative to the sample population, explicitly stated. Unless evident, then answer "unable to determine"
*Internal validity*	
8	If any of the results of the study were based on 'data dredging,' was this made clear? *Were any additional data analysis reported in the results not highlighted in the methodology?
9	Were statistical tests used to assess the main outcomes appropriate?
10	Were the main outcome measures accurate (valid and reliable)?

## Results

### Study Selection

The search strategy yielded a total of 4817 articles, of which 672 duplicates were removed. Subsequently, 4136 articles were excluded based on title and abstract screening. In total, 7 articles were included in this systematic review after full text screening and one additional article was included from backward citation tracking, resulting in a total of 8 studies being included. The most common reasons for exclusion of studies (i.e., based on titles and abstracts) were: wrong study field (97.3%), wrong population (1.7%), wrong outcome measures (0.6%), and wrong study design or statistical analyses (0.4%). In the second screening phase (i.e., based on full text articles), all further exclusions were due to a wrong study design or population (100%).

### Risk of Bias

The risk of bias assessment is presented in Table 4. Based on the modified assessment tool of Downs and Black [41], including only 10 items, we were unable to confirm the external validity of all the studies included due to the lack of information regarding the proportion of individuals recruited relative to the overall sample population. Furthermore, no internal validity bias was apparent, except for some studies that failed to report data on the validity and reliability of the outcome measures used. In general, total scores ranged between 5/10 and 9/10 for study methodological quality and risk of bias.

**Table 4:** Results of the risk of bias assessment for all included studies

Study	Modified Downs and Black checklist item number											Total score out of 10
	Reporting							External validity	Internal validity			
	1	2	3	4	5	6	7		8	9	10	
Beck et al. [49]	•	○	•	•	•	•	-		•	•	-	7
Blagrove et al. [36]	•	•	•	•	•	•	-		•	•	•	9
Joubert et al. [50]	•	•	•	•	•	•	-		•	•	•	9
Melo et al. [51]	•	•	•	•	•	•	-		•	•	-	8
Mo et al. [52]	•	•	•	•	•	•	-		•	•	-	8
Seminati et al. [47]	○	○	•	•	•	○	-		•	•	-	5
Stiffler-Joachim et al. [46]	•	•	•	•	•	•	-		•	•	-	8
Tabor et al. [48]	•	○	•	•	•	○	-		•	•	-	6

• = *yes*; ○ = *no*; - = *unable to determine*

### Study Characteristics

From all included studies, 37.5% were performed in Europe (UK, Italy, Poland) [36, 44, 45], 37.5% in North America (USA) [46–48], 12.5% in South America (Brazil) [49] and 12.5% in Asia (China) [50]. Furthermore, and except for one study published in 2013 [44], all studies were published from 2018 onwards.

### Sample Characteristics

Information regarding study characteristics is provided in Table 5. The 8 included studies represented a total of 181 participants, including 52% males (n = 94) and 48% females (n = 87). The mean age of study participants ranged between 17.1 years [36] and 42.6 years [44]. Across studies, 68% of the participants were competitive runners (54% female) [36, 44, 45, 47, 48, 50], 22% were recreational runners (36% female) [44, 46, 49, 50] and 10% were novice runners (33% female) [44, 50].

**Table 5:** Overview of study sample characteristics, outcome measures, equations for calculating asymmetry magnitude, inter-limb asymmetry magnitudes, and main results of the association between inter-limb asymmetry and running performance-related metrics

Study and country (ISO code)	Participant characteristics				Asymmetry test/metric measured	Running performance outcome measure(s)	Equations for calculating asymmetry magnitude	Magnitude of inter-limb asymmetry	Associations with running performance
	N	Age (years)	Training status						
Beck et al. (2018) [49] USA	*n* = 10 ♂: 6 ♀: 4	23 ± 6	Participants ran at least 3 times per week for minimum 30 min	Step time asymmetry Ground contact time asymmetry Stance average vertical ground reaction force asymmetry Peak braking ground reaction force asymmetry Peak propulsive ground reaction force asymmetry Leg stiffness asymmetry Peak vertical ground reaction force asymmetry		Metabolic power	Symmetry index = $\frac{{| \left( {{\text{tstep}}, 1 {-} {\text{tstep}}, 2} \right) }}{{ 0.5 \times \left( {{\text{tstep}}, 1 + {\text{tstep}}, 2} \right) | }} \times 100$	Step time: 0%, 7%; 14% and 21% Ground contact time: NA Stance average vertical ground reaction force: NA Peak braking ground reaction force: NA Peak propulsive ground reaction force: NA Leg stiffness: NA Peak ground reaction force: NA	Metabolic power vs. step time asymmetry: *r*^*2*^ = NA, *p* < 0.001, *Metabolic power* = *0.35* $\bullet$ *tstep SI* + *0.67* vs. ground contact time asymmetry:* β* = 0.78, *r*^*2*^ = NA, p = 0.04, *regression equation:* NA vs. stance average vertical ground reaction force asymmetry:* β* = 0.35, *r*^*2*^ = NA,* p* < 0.001, *regression equation:* NA vs. peak braking ground reaction force asymmetry:* β* = 0.13, *r*^*2*^ = NA, *p* < 0.001, *regression equation:* NA vs. peak propulsive ground reaction force asymmetry:* β* = 0.20, *r*^*2*^ = NA, *p* < 0.001, *regression equation:* NA vs. leg stiffness asymmetry:* β* = 0.39, *r*^*2*^ = NA, p = 0.04, *regression equation:* NA vs. peak vertical ground reaction force asymmetry: p = 0.47, *regression equation:* NA
Blagrove et al. (2021) [36] GBR	*n* = 31 ♂:15 ♀: 16	♂: 17 ± 1 ♀: 17 ± 1	Competitive middle and long-distance runners Non-strength trained	Lower limb extensor bilateral symmetry index Hip extension strength asymmetry Hip abduction strength asymmetry		Energy cost Best race performance	Symmetry index (%) = $\frac{{\left( {{\text{stronger }}\;{\text{limb }}{-}{\text{ weaker}}\;{\text{ limb}}} \right)}}{Total}{ } \times { }100$ Strength asymmetry (%) = $\frac{{\left( {{\text{Stronger}}\;{\text{ limb }}{-}{\text{ weaker}}\;{\text{ limb}}} \right)}}{{\text{stronger limb}}}{ } \times { }100$	Isometric quarter-squat strength: ♂: 8.3 ± 5.9% ♀: 8.6 ± 5.2% Hip extension strength: ♂: 4.6 ± 3.9% ♀: 7.4 ± 5.6% Hip abduction strength: ♂: 6.2 ± 4.8% ♀: 9.9 ± 5.9%	Males: - Lower limb extensor bilateral symmetry index vs. running economy: *r*_*s*_ = 0.30, *p* > 0.05 vs. race performance: *r*_*s*_ = -0.09, *p* > 0.05 - Hip extension strength asymmetry vs. running economy: *r*_*s*_ = 0.02, *p* > 0.05 vs. race performance: *r*_*s*_ = -0.26, *p* > 0.05 - Hip abduction strength asymmetry vs. running economy: *r*_*s*_ = -0.19, *p* > 0.05 vs. race performance: *r*_*s*_ = 0.05, *p* > 0.05 Females: - Lower limb extensor bilateral symmetry index vs. running economy: *r*_*s*_ = 0.30, *p* > 0.05 vs. race performance: *r*_*s*_ = -0.07, *p* > 0.05 - Hip extension strength asymmetry vs. running economy: *r*_*s*_ = 0.11, *p* > 0.05 vs. race performance: *r*_*s*_ = -0.20, *p* > 0.05 - Hip abduction strength asymmetry vs. running economy: *r*_*s*_ = 0.85, *p* < 0.001 vs. race performance: *r*_*s*_ = -0.47, *p* = 0.07
Joubert et al. (2020) [50] USA	*n* = 11 ♂: 7 ♀: 4	♂: 21 ± 1 ♀: 19 ± 1	NCAA Division I athletic program 1500 m, 10.000 m and 800 m specialists	Ground contact time		Caloric unit cost	Ground contact imbalance = $|\text{\% }t\text{left }-\text{ \% }t\text{right}|$	Ground contact time: NA	Ground contact time imbalance vs. caloric unit cost (kcal kg^−1^ km^−1^): *r* = 0.81, *r*^*2*^ = 0.66, *CI*: [0.37 – 0.93], *p* = 0.003 *Caloric Unit Cost* = *0.9649* + *0.0354 *$\bullet$* ground contact time*
Melo et al. (2020) [51] BRA	*n* = 13 ♂: 8 ♀: 5	36 ± 4	Amateur trained runners	Dynamical symmetry index in vertical, mediolateral en anteroposterior directions based on body center of mass displacements during a 10 km run		Mechanical efficiency	Global symmetry index = $\frac{dx \bullet \overline{SIx }+dy \bullet \overline{SIy }+dz \bullet \overline{SIz} }{dx+dy+dz}$	Global symmetry index: NA	Global symmetry index vs. Mechanical efficiency: *r* = 0.66, *r*^*2*^ = 0.43, *p* = 0.02, *Mechanical efficiency* = *2.4* + *31.2* $\bullet$ *global symmetry index*
Mo et al. (2020) [52] CHN	*n* = 31 ♂: 13 ♀: 18	Competitive runners: 32 ± 4 Recreational runners: 35 ± 7 Novice runner: 29 ± 4	Competitive, recreational and novice runners	Stride asymmetry Step asymmetry Stance asymmetry Flight time asymmetry Duty factor asymmetry		Fixed instrumented treadmill velocities (i.e., 8, 9, 10, 11 and 12 km/h)	Symmetry index = $\frac{{\left| {{\text{ Xright }} {-} Xleft} \right|{ }}}{{{ }0.5{\text{ X }}\left( {{\text{Xright}} + Xleft} \right){ }}}{\text{X}}$	Flight time: 5.0 ± 4.1% Time to peak vertical ground reaction force: 7.1 ± 3.2% Vertical average loading rate: 17.3 ± 7.7%	Running velocity vs. flight time asymmetry:* p* = 0.01 - Competitive runners: *r*^*2*^ = 0.95, *SI* = *-0.7 *$\bullet$* Velocity* + *10.5* - Recreational runners: *r*^*2*^ = 0.95, *SI* = *0.5* $\bullet$ *Velocity*^*2*^ + *10.2* $\bullet$ *Velocity* + *57.9* - *Novice runners: r*^*2*^ = 0.64, *SI* = *0.5* $\bullet$ *Velocity*^*2*^ + *10.0* $\bullet$ *Speed* + *57.3* vs. time to peak vertical ground reaction force asymmetry: *p* = 0.03 - Competitive runners:* r*^*2*^ = 0.95, *SI* = *-0.5 *$\bullet$* Velocity* + *112.6* - Recreational runners: *r*^*2*^ = 0.99, *SI* = *0.3* $\bullet$ *Velocity*^*2*^* – 6.1* $\bullet$ *Speed* + *35.4* - Novice runners*: r*^*2*^ = 0.78, *SI* = *0.6* $\bullet$ *Velocity* + *0.9* vs. vertical average loading rate asymmetry: *p* = 0.002 - Competitive runners:* r*^*2*^ = 0.94, *SI* = *-2.4* $\bullet$ *Velocity* + *40.9* - Recreational runners: *r*^*2*^ = 0.88, *SI* = *1.9* $\bullet$ *Velocity*^*2*^* – 37.9* $\bullet$ *Velocity* + *203.4* - Novice runners: *r*^*2*^ = 0.93, *SI* = *0.3* $\bullet$ *Velocity*^*2*^* – 5.0* $\bullet$ *Velocity* + *40.3*
Seminati et al. (2013) [47] ITA	*n* = 19 ♂: 19 ♀: 0	Untrained runners: 33 ± 13 Occasional runners: 32 ± 12 Skilled runners: 43 ± 7	Untrained, occasional and skilled runners	Anatomical symmetry (i.e., volumes of pelvis district, upper-leg district, lower-leg district and global anatomical cross correlation value) Dynamical symmetry: body center of mass trajectory (SI^x^, SI^y^, SI^z^, GI)		Metabolic cost	Symmetry degree between 3D split volumes: $r_{i,j,k} = \frac{{\mathop \sum \nolimits_{x,y,z} \left[ {Rv\left( {x,y,z} \right) - \overline{{Rv_{i,j,k} }} } \right] \cdot \left[ {Lrv\left( {x - i,y - j,z - k) - \overline{Lrv} } \right) {-} \overline{{Rv_{i,j,k} }} } \right]}}{{\sqrt {\mathop \sum \nolimits_{x,y,z} \left[ {Rv\left( {x,y,z} \right) - \overline{{Rv_{i,j,k} }} } \right]^{2} \cdot \sum x,y,z \left[ {Lrv\left( {x - i,y - j,z - k} \right) - \overline{Lrv} } \right]^{2} } }}$ Global symmetry index = $\frac{{dx \cdot \overline{SIx} + dy \cdot \overline{SIy} + dz \cdot \overline{SIz} }}{dx + dy + dz}$	Anatomical symmetry: Pelvis district: 0.77 ± 0.09 Upper-leg district: 0.82 ± 0.05 Lower-leg district: 0.83 ± 0.05 Global anatomical cross correlation value: NA Dynamical symmetry: *SI*^x^:: 0.72 ± 0.06 *SI*^y^: 0.88 ± 0.04 *SI*^z^: 0.85 ± 0.05 GI: NA	Metabolic cost vs. anatomical symmetry: - Pelvis district: *r* = 0.16, *p* = 0.55 - Upper-leg district: *r* = -0.11, *p* = 0.66 - Lower-leg district: *r* = 0.06, *p* = 0.82 - Global anatomical cross correlation value: *r* = 0.07,* R*^*2*^ = 0.22, *p* = 0.06 Global symmetry index = *0.460 *$\bullet$* Global anatomical cross correlation* + *0.340* vs. dynamical symmetry: - *SI*^x^: *r* = 0.01, *p* = 0.98 - *SI*^y^:* r* = 0.11, *p* = 0.67 - *SI*^z^:* r* = 0.21, *p* = 0.39 - GI: *r* = -0.001, *p* = 0.995
Stiffler-Joachim et al. (2021) [46] USA	*n* = 54 ♂: 26 ♀: 28	♂: 19 ± 1 ♀: 19 ± 1	Collegiate cross-country runners	Ground contact time Vertical ground reaction force Average loading rate Braking impulse Propulsive impulse Foot inclination angle Peak hip flexion Peak hip extension Peak knee flexion Peak ankle dorsiflexion Peak hip adduction Peak pelvic drop Base of gate		Within-season personal records (i.e., 8 km for male runners and 6 km for female runners)	Kinematic outcomes: $\left| {{\text{ Xright }} {-} Xleft} \right|$ Kinetic outcomes: Symmetry index = $\frac{{\left| {{\text{ Xright }} {-} Xleft} \right|}}{{{ }0.5{ } \times { }\left( {{\text{Xright}} + Xleft} \right){ }}} \times {\text{X}}$	Ground contact time: 1.7%—2.3% Vertical ground reaction force: 2.6%—3.1% Average loading rate: 13.8%—19.7% Braking impulse: 8.1%—11.1% Propulsive impulse: 3.1%—8.4% Foot inclination Angle: 2.2°—3.2° Peak hip flexion: 1.1°—2.0° Peak hip extension: 1.4°—1.9° Peak knee flexion: 2.4°—3.1° Peak ankle dorsiflexion: 2.6°—3.2° Peak hip adduction: 2.6°—3.3° Peak pelvic drop: 1.8°—2.7° Base of gate: 1.1 cm – 1.4 cm	Personal records vs. ground contact time asymmetry: *β* = -4.50, *CI*: [-14.9, 6.3], *r*^*2*^*:* NA, *p* = 0.39, *regression equation:* NA vs. vertical ground reaction force asymmetry: *β* = -2.70, *CI*: [-9.2, 3.7], *r*^*2*^ = NA, *p* = 0.42, *regression equation:* NA vs. average loading rate asymmetry: *β* = -3.30, *CI*: [-8.1, 1.4], *r*^*2*^ = NA, *p* = 0.17, *regression equation:* NA vs. braking impulse asymmetry: *β.* = 0.00 *CI*: [-10.4, 10.9], *p* = 0.99, *regression equation:* NA vs. propulsive impulse asymmetry: *β* = 14.60, *CI*: [4.4, 25.0], *r*^*2*^ = NA, *p* < 0.01, *regression equation:* NA vs. foot inclination angle asymmetry: *β* = -3.90; *CI*: [-10.9, 2.9],* r*^*2*^ = NA, *p* = 0.27, *regression equation:* NA vs. peak hip flexion asymmetry: *β* = -4.10, *CI*: [-14.4, 5.7], *r*^*2*^ = NA, *p* = 0.41, *regression equation:* NA vs. peak hip extension asymmetry: *β* = -3.10, *CI*: [-13.7, 7.2], *r*^*2*^ = NA, *p* = 0.56, *regression equation:* NA vs. peak knee flexion asymmetry: *β* = -1.30, *CI*: [-7.2, 4.3], *r*^*2*^ = NA, *p* = 0.63, *regression equation:* NA vs. peak ankle dorsiflexion asymmetry: *β* = -6.10, *CI*: [-12.9, 0.7], *r*^*2*^ = NA, *p* = 0.08, *regression equation:* NA vs. peak hip adduction asymmetry: *β* = 0.40; *CI*: [-5.5, 6.1], *r*^*2*^ = NA, *p* = 0.90, *regression equation:* NA vs. peak pelvic drop asymmetry: *β* = -2.80, *CI*: [-4.3, 9.7], *r*^*2*^ = NA, *p* = 0.42, *regression equation:* NA vs. base of gate asymmetry: *β* = -5.50, *CI*: [-18.9, 7.8], *r*^*2*^ = NA, *p* = 0.43, *regression equation:* NA
Tabor et al. (2019) [48] POL	*n* = 12 ♂: 0 ♀: 12	Group A (athletes with at least 7 years of training experience): 23 ± 3 Group B (athletes with at least three years of training): 23 ± 1	Intermediate and advanced middle-distance runners	Muscle strength symmetry Support phase time symmetry Swing phase time symmetry		Running velocity	Symmetry index = $\frac{2 \times (\text{right}-\text{left}) }{(\text{right}+\text{left})}$ Asymmetry index = $\underset{{t=t}_{1}}{\overset{{t}_{2}}{\int }}A|{x}_{r}\left(t\right)- {x}_{l}\left(t\right)|\text{dt}$	Group A: Muscle strength: 6.6 ± 6.0% Support phase time: 7.4 ± 9.2% Swing phase time: 4.0 ± 3.1% Group B: Muscle strength: 4.2 ± 2.1% Support phase time: 8.6 ± 3.7% Swing phase time: 6.0 ± 3.6%	Running velocity vs. muscle strength symmetry: *β* = -5.77, *r*^*2*^ = NA, *p* = 0.01, *regression equation:* NA vs. support phase time symmetry: *β* = -6.64, *r*^*2*^ = NA, *p* = 0.03, *regression equation:* NA vs. swing phase time symmetry: *β* = -2.47, *r*^*2*^ = NA, *p* > 0.05, *regression equation:* NA

♂ = male(s), ♀ = female(s), NA = not available, CI = confidence interval, *β* = beta value, *r* = Pearson correlation coefficient, *r*_*s*_ = Spearman rank order correlation coefficient, *r*^*2*^ = R-squared value, t = time; dx, dy and dz = vector displacement, SI = symmetry index for three directions (x, y and z); x anteroposterior direction, y = mediolateral direction, z = vertical direction, GI = global symmetry index, Xright = value of the right leg; Xleft = value of the left leg*,* ${r}_{i,j,k}$ = normalised cross-correlation coefficient, $\overline{Lrv }$ = voxel mean value of the left volume, $\overline{{Rv }_{i,j,k}}$ = voxel mean value of the right volume, ${x}_{r}\left(t\right)$ = value of specific variable recorded for the right leg at time t, ${x}_{r}\left(t\right)$ = value of specific variable recorded for the left leg at time t

### Test and Outcome Measures

#### Asymmetry Tests and Metrics

Two studies examined functional asymmetry [36, 45], 1 study morphologic asymmetry [44], 6 studies kinematic asymmetry [45–50] and 2 studies kinetic asymmetry [46, 48] in relation to middle- and long-distance running performance and/or its determinants. Functional asymmetry was assessed using strength tests (i.e., isometric quarter-squat, isometric hip extension, isometric hip abduction, isokinetic knee flexion, isokinetic knee extension) [36, 45], whilst morphological asymmetry was measured by performing magnetic resonance imaging [44]. Kinematic (e.g., spatiotemporal variables: step time, ground contact time, flight time, stride asymmetry; displacements in body centre of mass) and kinetic (e.g., ground reaction force, leg stiffness, braking impulse) variables were all collected while running [46–50] or during the execution of a CMJ [45]. Kinematic data were measured using an instrumented treadmill in 2 studies [46, 50], an accelerometer in a wrist wearable in 1 study [47], a 3D accelerometer in 1 study [49], a passive marker system in 1 study [48], an inertial measurement unit (IMU) in 1 study [45] and an optoelectronic system in 1 study [44].

#### Equations for Calculating Asymmetry

A variety of equations were used to express the magnitude of inter-limb asymmetry among participants in the 8 included studies (see Table 5). Four studies calculated the percentage of asymmetry related to the right versus left lower limb [45, 47, 48, 50], while only one accounted for stronger and weaker lower limb in the formula [36]. Furthermore, two studies used the global symmetry index to identify the magnitude of asymmetry in body centre of mass trajectory [44, 49], whereas one study also adopted a normalised cross-correlation coefficient to quantify the magnitude of asymmetry between 3D split volumes with magnetic resonance [44].

#### Running Performance-Related Metrics

The middle- and long-distance running performance variables taken into account could be divided in two specific subcategories: determinants of running performance versus actual running performance metrics based on race performances. As an important determinant of running performance, running economy (e.g., metabolic power [46], energy cost [36], caloric unit cost [47], metabolic cost [44] and mechanical efficiency [49]) was examined. All of these physiological parameters were examined in a steady metabolic state. Furthermore, race performances or personal best times [36, 48] and running velocity [45, 50] were used as an actual running performance metric in four included studies. Metabolic power, metabolic cost, energy cost, caloric unit cost and personal best times should be interpreted inversely with a view to running performance, as lower values in these metrics correspond to better running performance.

### The Association Between Inter-limb Asymmetry and Running Performance-Related Metrics

Evidence for an association between inter-limb asymmetry and middle- and long-distance running performance (and/or its determinants) in healthy populations was mixed. All asymmetry outcomes could be sub-divided into four dimensions (i.e., functional asymmetry, morphologic asymmetry, kinematic asymmetry and kinetic asymmetry) and were assessed independently in view of their link with running performance metrics. Table 6 summarises all (significant positive, significant negative or no significant) associations between functional, morphological, kinematic and kinetic asymmetry and running performance-related metrics.

**Table 6:** Summary of the (significant positive and significant negative or not significant) associations between inter-limb asymmetry and running performance-related metrics

Type of asymmetry	# of asymmetry outcome measures associated with running performance		Studies
Functional asymmetry	Significantly positive	0	–
	Significantly negative	3	[36, 50]
	Not significant	10	[36]
Morphological asymmetry	Significantly positive	0	–
	Significantly negative	0	–
	Not significant	4	[49]
Kinematic asymmetry	Significantly positive	1	[47]
	Significantly negative	6	[46, 48, 50–52]
	Not significant	13	[47, 50]
Kinetic asymmetry	Significantly positive	0	–
	Significantly negative	7	[47, 48, 51]
	Not significant	3	[47, 51]
Overall	Significantly positive	1	[47]
	Significantly negative	16	[36, 46–48, 50–52]
	Not significant	30	[47, 49, 51]

Running performance includes actual running performance as well as running performance determinants. The outcome measures from the determinants metabolic power, metabolic cost, energy cost, caloric unit cost and personal best times were inversely interpreted given their negative relationship with running performance. Similarly, symmetry magnitudes were inversely construed as asymmetry magnitudes

#### Functional Asymmetry Linked to Running Performance-Related Metrics

The magnitude of functional inter-limb asymmetry across studies ranged between 4.2 and 9.9% [36, 45]. Tabor et al. [45] reported significant negative associations of asymmetry in the sum of muscle torque under static conditions in the hip, knee and ankle with maximal running velocity (*β* = − 5.77, *p* < 0.01). In contrast, swing phase time symmetry measured during a CMJ was not significantly correlated with running velocity (*β* = − 2.50, *p* > 0.05) [45]. In the study by Blagrove et al. [36], negligible associations were reported between muscle strength asymmetry and race performance as well as running economy (race performance: *r* = − 0.20 to 0.13; running economy: *r* = 0.02 to 0.30), except for the correlations found between hip abduction strength asymmetry and race performance (*r* = -0.47, *p* = 0.07) as well as running economy (*r* = 0.85, *p* < 0.001) in female middle- and long-distance runners.

#### Morphological Asymmetry Linked to Running Performance-Related Metrics

The only study documenting morphological asymmetry included in this systematic review reported no significant associations of anatomical asymmetry (i.e., volume assessed by means of magnetic resonance images) at the pelvis and lower limb level with the metabolic cost of running (*r* = 0.06–0.16, *p* = 0.55–0.82) [44]. In the latter study, the range of morphological asymmetry was described in absolute terms, and ranged between 0.77 and 0.83 [44].

#### Kinematic Asymmetry Linked to Running Performance-Related Metrics

Except for one study in which the magnitude of step time inter-limb was predetermined while running on a treadmill and set at 0%, 7%; 14% and 21%, respectively, the magnitude of kinematic asymmetry ranged between 1.7 and 8.6% [45–50]. Significant associations were reported between kinematic asymmetry and metabolic power (*β* = 0.10–0.80, *p* < 0.001) [46]. More specifically, for every 10% increase in step time asymmetry and ground contact time asymmetry an increase of 3.5% and 7.8% in metabolic power was observed, respectively [46]. Similarly, ground contact time asymmetry was strongly and positively related to caloric unit cost (*r* = 0.81, *p* = 0.003) [47]. For every 1% increase in ground contact time asymmetry, the caloric unit cost increased by 0.0354 kcal kg^−1^ km^−1^. Symmetry in displacements of the body centre of mass during running was moderately and positively related to mechanical efficiency (*r* = 0.66, *p* = 0.015) but not associated with metabolic cost (*r* = − 0.00, *p* = 0.995) [44, 49]. Kinematic asymmetry was not related to within-season personal best times (*β* = − 5.50 to 0.40, *p* = 0.27–0.90) [48], with the exception of asymmetry in peak ankle dorsiflexion (i.e., for every 1° increase in peak ankle dorsiflexion asymmetry, personal best times over 8 km for male runners and 6 km for female runners decreased by 7.6 s). Support phase time asymmetry was negatively related to running velocity (*β* = − 6.60, *p* = 0.03) [45].

#### Kinetic Asymmetry Linked to Running Performance-Related Metrics

The magnitude of kinetic inter-limb asymmetry across studies ranged between 2.6% and 19.7%. It was reported that every 10% increase in peak braking ground reaction force asymmetry (*β* = 0.13, *p* < 0.001), peak propulsive ground reaction force asymmetry (*β* = 0.20, *p* < 0.001), stance average vertical ground reaction force (*β* = 0.35, *p* < 0.001) and leg stiffness asymmetry (*β* = 0.39, *p* = 0.04), respectively, elicits a 1.3%, 2.0%, 3.5% and 3.9% metabolic power increase [46]. In contrast, peak vertical ground reaction force asymmetry was not found to be significantly correlated with net metabolic power (*p* = 0.47) and within-season personal best times (*β* = − 2.70, *p* = 0.42) [46, 48]. Conversely, peak vertical ground reaction force asymmetry while running was reported to be significantly related to running velocity (i.e., 3 min at fixed velocity of 8, 9, 10, 11 and 12 km/h) (*p* = 0.03; competitive runners: *β* = − 0.50, recreational runners: *β* = 0.30, novice runners: *β* = 0.60) [50]. In competitive and recreational runners, asymmetry in peak vertical ground reaction force and vertical load rate asymmetry showed a linear and U-shaped trend across velocities, respectively [50]. In novice runners, a lower asymmetry of time to peak vertical ground reaction force was associated with increasing running velocity, whilst asymmetry in vertical load rate did not differ across velocities [50]. As opposed to average loading rate and braking impulse asymmetry (*β* = 0.00, *p* = 0.99), propulsive impulse asymmetry (*β* = 14.60, *p* < 0.01) was positively associated with within-season personal best times on distances of 8 km for male runners and 6 km for female runners [48]. Controlled for sex, for every 5% increase in propulsive impulse asymmetry, personal best times within the running season increased by 16 s [48].

## Discussion

The main objective of this systematic review was to synthesize and evaluate the available literature regarding the associations between inter-limb asymmetry at lower limb level and middle- and long-distance running performance-related metrics in healthy populations. According to the risk of bias assessment, all included studies were of moderate to strong quality. To compare and evaluate the association of inter-limb asymmetry with running performance (and/or its determinants), it was necessary to differentiate between dimensions to quantify asymmetry. Therefore, this review addressed the link between functional, morphological, kinematic and kinetic inter-limb asymmetry with running performance-related metrics, separately. Overall, the results from this review indicate that functional (3 out of 13 metrics), kinematic (6 out of 20 metrics) and kinetic (7 out of 10 metrics) inter-limb asymmetry were partly negatively associated with running performance (and/or its determinants). In contrast, no significant association was observed between morphological asymmetry and the determinants of running performance. It is important to note that the limited available literature on the topic alongside the high heterogeneity in terms of asymmetry assessments and running metrics, as well as the different mathematical equations for calculating asymmetry magnitude, made it difficult to compare studies and impossible to conduct a meta-analysis. This discrepancy across test protocols and outcome measures resulted in inconsistent findings highlighting the task, metric, test occasion and inter- and intra-individual specific nature of inter-limb asymmetry and its magnitude [7–13, 51–53].

### Functional Asymmetry and Running Performance-Related Metrics

Functional asymmetry was most commonly assessed using strength measures (e.g., isometric strength) [36, 45]. This is unsurprising since strength training-induced neuromuscular adaptations have been demonstrated to enhance running economy (i.e., 2—8%) as well as time trial performance and maximal sprint velocity in middle and long-distance runners [54–56]. Moreover, larger magnitudes of inter-limb strength asymmetry have also been associated with increased gait asymmetry (*r* = 0.44), indicating a transfer from functional assessments to sport-specific measures [57].

Blagrove et al. [36] examined the relationship between the magnitude of isometric muscle strength asymmetry (i.e., quarter-squat, hip extension and hip abduction) and running performance as well as running economy (i.e., energy cost in kJ^.^kg^−75.^km^−1^) in male and female competitive middle- and long-distance runners. In general, this study observed group mean asymmetry values ranging between 4.6–8.4%, resulting in negligible associations between inter-limb strength asymmetry and running performance (*r* =− 0.26 to 0.13), and inter-limb strength asymmetry and running economy (*r* =− 0.02 to 0.13). However, a larger magnitude of inter-limb asymmetry of 10% was found in hip abduction torque asymmetry for the female middle- and long-distance runners. This inter-limb asymmetry in abduction strength was significantly positively correlated (*r* = 0.85) with running economy, indicating the potential negative impact of larger inter-limb asymmetry magnitudes on running performance as higher energy costs are detrimental to middle- and long-distance performances [58]. Similarly, Tabor et al. [45] reported that reductions in the magnitude of the sum of muscle torque in knee and hip flexors and extensors were negatively correlated with running velocity in female middle-distance runners (*β* = − 6.64). However, given the task-dependent nature of functional asymmetry [7], it may not be advisable to add together different strength measures to determine strength asymmetry. Therefore, this latter result should be interpreted with caution.

### Morphological Asymmetry and Running Performance-Related Metrics

Previous research documented a negative relationship between asymmetry in various traits (e.g., nostrils and ears) and running performance in middle-distance runners [59]. However, the existing literature on the association between the magnitude of morphological asymmetry and (determinants of) running performance-related metrics seems to be limited to only one study in untrained, occasional and skilled runners. A first important finding of this study conducted by Seminati et al. [44] was the moderate and positive correlation (*r* = 0.61) between anatomical asymmetry (i.e., side-to-side differences in volume of the lower limbs measured by magnetic resonance imaging) and dynamical asymmetry (i.e., body centre of mass displacements), indicating that runners with greater magnitudes of morphological asymmetry tend to exhibit more pronounced asymmetrical running patterns. Moreover, this latter study showed that training status moderated this relationship, as more experienced runners showed smaller magnitudes of dynamic asymmetry at higher running velocities compared to their untrained peers. However, this study did not report a significant correlation between anatomical asymmetry and metabolic cost. The authors speculated that certain physiological adaptations may compensate for the relatively small anatomical asymmetry magnitudes observed (i.e., 0.77–0.83%), regardless of training status [47].

Given the scarcity of literature on the link between morphological inter-limb asymmetry and running performance, it is difficult to draw clear conclusions. However, it is important to note that none of the studies included in this review addressed leg length discrepancies. This is probably because leg length differences are typically reported as an absolute difference between the right and left lower limb, rather than as a relative asymmetry score (i.e., expressed as a percentage). As such, larger absolute leg length differences (> 2 cm) have been reported to increase oxygen consumption and energy expenditure during walking [60, 61]. Moreover, and although this seems to be individual specific, absolute leg length differences have been positively associated with a more pronounced gait asymmetry (*r* = 0.29–0.51) [62, 63]. In contrast, leg length differences smaller than 1 cm do not appear to be significantly associated with running economy [47, 64]. These results support the notion that the magnitude of morphological asymmetry between the lower limbs could affect running performance-related metrics [62].

### Kinematic Asymmetry and Running Performance-Related Metrics

Despite the wide range of kinematic asymmetry magnitudes observed (i.e., 3–54%), the narrative review by Carpes et al. [16] concluded in 2010 that the available studies failed to establish significant relationships between kinematic asymmetry and running performance. Due to the recently growing interest in the topic, several studies attempted to investigate the association between kinematic inter-limb asymmetry and determinants of running performance as well as personal best times [45–47, 49, 50]. For instance, two studies included in the current systematic review indicated that inter-limb asymmetry in ground contact times (i.e., the average time each foot spends in contact with the ground while running) was correlated with impaired caloric unit cost (*r* = 0.81) and metabolic power (i.e., energy cost, based on O_2_ consumption and CO_2_ production [65]) (*β* = 0.78) [46, 47]. As discussed in a recent review by Moore et al. [66], there is still debate on whether short or long contact times are favourable in terms of running performance. Whereas short ground contact times are suggested to impose a higher metabolic cost due to the need for faster force production [67, 68], longer ground contact times are suggested to increase the metabolic cost during the increased deceleration, resulting in a lengthened braking phase [69]. However, the findings in our review indicate that inter-limb asymmetry in ground contact times has a negative impact on running economy, potentially impairing running performance. Similarly, step time asymmetry (i.e., including both the ground contact time and the subsequent aerial time) was significantly positively correlated (*β* = 0.35) with metabolic power [46]. This result is consistent with previous research in which larger asymmetric step times were associated with increased metabolic power in walking [70]. Beck et al. [46] attributed these findings to reduced mechanical energy conservation in asymmetric step times, resulting in increased muscle mechanical work per step and an increased metabolic rate.

Studies investigating the association of asymmetry in trajectories of body centre of mass and (determinants of) running performance revealed equivocal results. Whilst asymmetry in body centre of mass displacements was moderately negatively related to mechanical efficiency (*r* = − 0.66), no significant association was found with metabolic cost [44, 49]. Differences in duration of the running protocol have been postulated as a possible explanation for these discrepancies. Melo et al. [49] argued that longer distance protocols (e.g., 10 km) are more suitable for detecting kinematic asymmetry, which may not be evident in shorter running bouts. Moreover, variations in running experience [71], running intensity [72, 73] and muscle fatigue [74] could also explain these differences in findings.

Previous research showed that peak ankle dorsiflexion (i.e., maximal ankle dorsiflexion angle during stance phase) later in stance was positively related to running economy and thus possibly affected running performance [75]. In the study by Stiffler-Joachim et al. [48], inter-limb asymmetry in peak ankle dorsiflexion was the only kinematic variable that was significantly and negatively correlated with within-season personal best times (*β* = − 6.1, 95% CI [− 12.9, 0.7]). Every 1° increase in peak ankle dorsiflexion asymmetry was related to a 7.6 s decrease in the best running time over 8 km for male and 6 km for female distance runners. Whilst the underlying mechanism for this finding is unclear, it should be noted that the magnitude for peak dorsiflexion was quantified as the absolute value of the inter-limb differences and not as a percentage. Lastly, Tabor et al. [45] demonstrated that swing phase asymmetry can impair running velocity in intermediate and advanced middle-distance runners (i.e., 800–1500 m). Given that a shorter support phase has been related to increased running velocity [69, 76], it seems plausible that asymmetry in the extension of the swing phase could impair running velocity [45].

### Kinetic Asymmetry and Running Performance-Related Metrics

Stiffler-Joachim et al. [48] documented varying kinetic asymmetry percentages in National Collegiate Athletic Association (NCAA) Division I runners, ranging from 3% for peak vertical ground reaction force up to 20% for average vertical loading rate. Propulsive impulse asymmetry has been reported to be related to impaired race performance in distance running [48]. Given that a larger propulsive impulse (i.e., body acceleration) is related to increased energy consumption and thus higher metabolic cost, practitioners should also consider minimizing side-to-side differences in this respect. In contrast, inter-limb asymmetries in average vertical loading rate, braking impulse and peak vertical loading rate were not found to be significant predictors of race performance [66]. This could potentially be attributed to the fact that these associations were investigated in elite runners, who exhibited low overall asymmetry scores for these particular metrics.

Regarding the relationship between kinetic inter-limb asymmetry and determinants of running performance, the results indicated that stance average vertical ground reaction force, peak propulsive ground reaction force and leg stiffness asymmetry were positively associated with metabolic power in recreational runners [46]. This suggests that more pronounced kinetic inter-limb asymmetry could increase energy expenditure while running and thus potentially have an adverse effect on running performance. [46]. In contrast, peak ground reaction force asymmetry was not found to be significantly associated with metabolic power, demonstrating the variable nature of asymmetry [46]. This variability in asymmetry metrics and their associations with running performance-related metrics was further emphasized in the study conducted by Mo et al. [50]. The latter study indicated that the association between inter-limb kinematic asymmetry and running performance depends on the velocity of the running test, the running experience of the participants and the parameter of interest assessed [50]. Consistent with previous research, the magnitude of asymmetry not only varied considerably within kinetic variables, but also appeared to be more pronounced compared to kinematic variables [50, 77].

### Limitations and Strengths

Although this is the first systematic review to provide a holistic view on the available evidence concerning lower inter-limb asymmetry and the association with (determinants of) running performance in middle- and long-distance runners, this research effort is not without limitations.

First, it is difficult to draw definitive conclusions due to the high heterogeneity among the included studies. More specifically, the heterogeneity was evident in a variety of dimensions, (e.g., functional, morphological, kinematic or kinetic), methods, equations and metrics being used to assess and express inter-limb asymmetry, as well as in a diversity of population characteristics (e.g., sex, age and/or training status of participants). Furthermore, caution is warranted when interpreting these results because of the scarcity of eligible studies, making comparisons between study results difficult and less robust. It is also important to note that associations between inter-limb asymmetry and determinants of running performance do not necessarily indicate a (causal) relationship between inter-limb asymmetry and running performance. Moreover, only a quarter of the studies documenting Pearson’s or Spearman’s rank order correlations also reported an assessment of normality on their raw data. Since falsely (i.e., with non-normal data) using a Pearson’s correlation coefficient highly increases type I error rates, justification for the use of parametric statistics by means of normality tests (e.g., Shapiro–Wilk test) is essential [78]. Furthermore, the small sample sizes frequently employed limit the statistical power of the studies, making it difficult to establish meaningful correlations [79]. Lastly, only one study reported the reliability of the asymmetry metric of interest. Given the inherently high variable nature of inter-limb asymmetry [14], a good test–retest as well as inter-rater reliability are necessary to ensure the quality of the data.

### Directions for Future Research

**Fig. 2:**
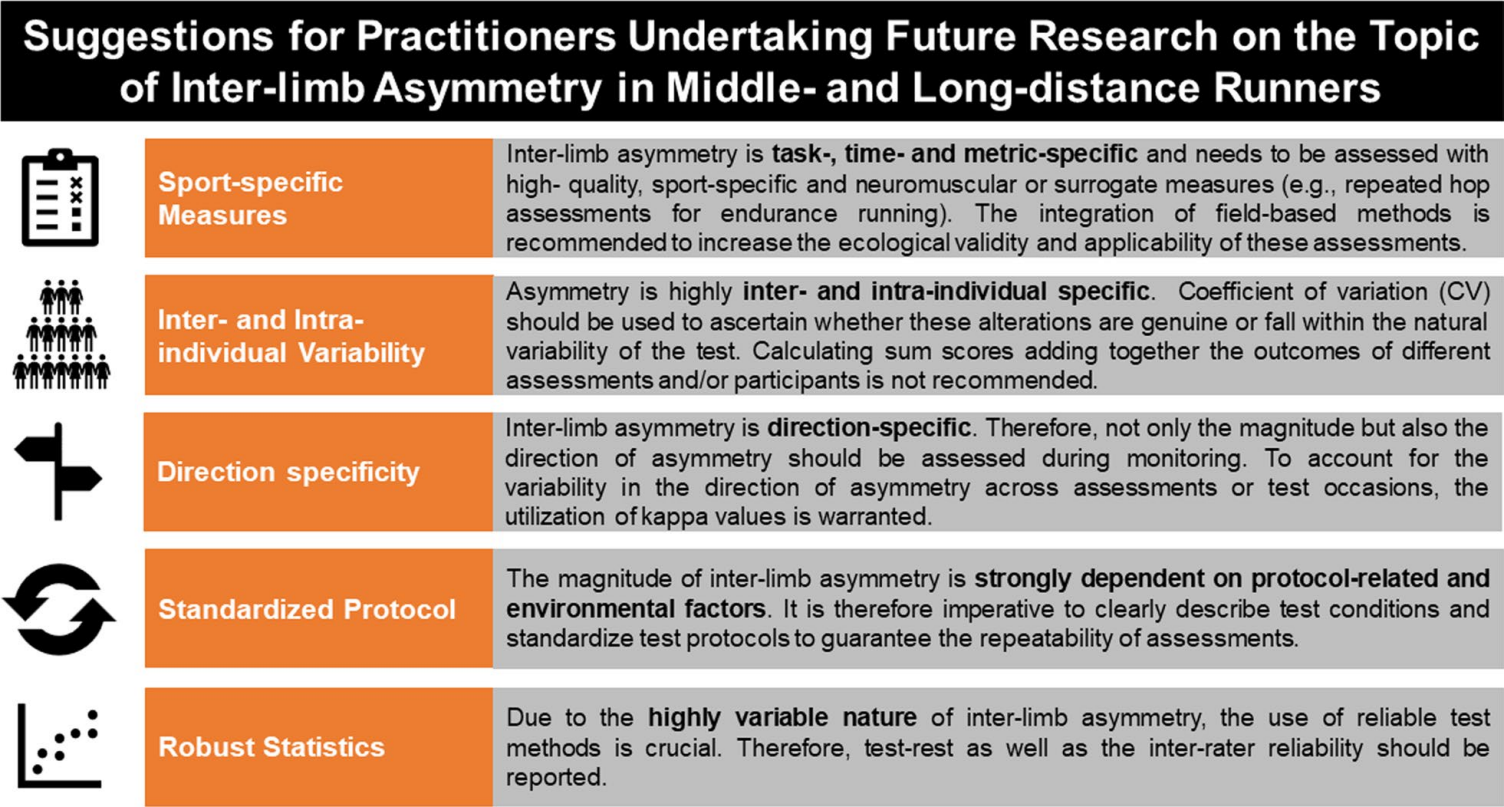
Points of attention for future researchers of inter-limb asymmetry in endurance runners

By analogy with the work of Afonso et al. [14], the synopsis of literature on the topic presented in the current systematic review is important to identify a research agenda highlighting some key areas for future research on inter-limb asymmetry in middle- and long-distance runners (see Fig. 2 for an illustrative overview):

1. In literature, inter-limb asymmetry is often not reported using sport-specific and field-based assessments in middle- and long-distance runners. Whilst functional asymmetry is generally measured using maximal (isometric) strength, (repeated) hop tests are presumed to have a greater ecological validity for assessing inter-limb asymmetry in runners due to their ability to measure various facets related to the stretch–shortening cycle [80]. Notably, storing and returning mechanical energy in the process of elastic energy utilization plays a key role in the metabolic energy-saving mechanism, and consequently running economy [81]. In this regard, leg stiffness (i.e., resistance to deformation of the limb) and reactive strength (i.e., the ability to effectively use the stretch and shortening cycle as well as the energy produced by the muscle–tendon complex) have been proposed to be important neuromuscular factors contributing to the elastic energy utilization [66, 82, 83]. Given that these factors can be measured using (repeated) hop tests and rebound jump protocols, significant correlations (*r* = 0.58–0.70) between countermovement jump metrics and running economy have been previously reported [84]. Hence, future research should consider investigating the effect of functional inter-limb asymmetry in leg stiffness and reactive strength using unilateral (repeated) hop tests. Moreover, the use of sport-specific, valid and reliable field-based assessments of functional asymmetry is needed to enhance the ecological validity and applicability among practitioners.
2. Disparities in asymmetry outcomes and magnitudes between different types of runners underscore the necessity for practitioners to account for inter-individual differences. Variables such as type of running (e.g., track versus road running), training status of runners (e.g., trained versus untrained) and injury history of runners (i.e., injured versus non-injured) should be considered when assessing lower inter-limb asymmetries [50, 85–87]. Although larger magnitudes of inter-limb asymmetry are expected in novice middle- and long-distance runners compared to elite middle- and long-distance runners [50], the results of the present review indicate that – based on the included studies – 68% of research has been conducted in competitive runners and only 22% in recreational runners and 10% in novice runners. Therefore, addressing a more diverse range of running populations in terms of training status, age and sex, while acknowledging the high inter- and intra-variability, is warranted.
3. The direction of asymmetry is highly variable between tasks and between test occasions [88, 89]. For instance, a distance runner may favour their right limb on a first test occasion whilst favouring their left limb on a second test occasion. Given that asymmetry is a ratio metric, reporting kappa values is highly recommended to assess differences in the direction of asymmetry between different tasks and/or test occasions.
4. Several factors relating to test protocols, such as running velocity, test intensity or fatigue, will likely induce intra-individual differences in asymmetry [50, 51, 72]. In addition, environmental factors such as the running surface, air humidity and ambient temperature may possibly lead to different asymmetry magnitudes and/or running performances. This accentuates the need for a standardized approach under stable conditions when evaluating inter-limb asymmetry.
5. Recognizing the highly variable nature of inter-limb asymmetry, researchers are urged to report the reliability of their tests and related outcome measures (e.g., test–retest or inter-rater reliability) to mitigate the impact of fluctuations on asymmetry due to test errors. A standardized approach for expressing asymmetry magnitude across studies is also needed, preferably using “stronger” and “weaker” limb instead of “right” and left “limb” [90].

## Conclusion

In summary, the limited literature on inter-limb asymmetry in middle- and long-distance runners displayed a high heterogeneity regarding study samples, study methods, assessments and metrics used, making direct comparisons difficult. With the exception of one study demonstrating a positive association between inter-limb asymmetry and running performance, the majority of findings suggest inter-limb asymmetries are either negatively associated with or do not affect running performance or its determinants in healthy populations. However, more research across diverse running populations is needed to confirm these assertions and to establish critical thresholds in this regard. Practitioners should be mindful of the task, test occasion and metric specificity as well as the inter- and intra-individual variability when monitoring inter-limb asymmetry.

## Data Availability

Not applicable.

## Abbreviations

CMJ: Countermovement jump
DJ: Drop jump
PRISMA: Preferred Reporting Items for Systematic reviews and Meta-Analyses
PICO acronym: Population (P), outcome (O) and study design (S)
